# Letter to the Editor: Integrating oral microbiome biomarkers into global disease burden assessments to enhance early-onset type 2 diabetes prevention strategies

**DOI:** 10.1097/JS9.0000000000004750

**Published:** 2026-01-13

**Authors:** Chenglu Ruan, Xiang Zheng, Lin Wang

**Affiliations:** a Department of Stomatology, Sanming Integrated Medicine Hospital, Fujian, China; b School of Artificial Intelligence in Traditional Chinese Medicine, Shanghai University of Traditional Chinese Medicine, Shanghai, China; c Department of Endocrinology, Sanming Integrated Medicine Hospital, Fujian, China; d Department of Product Design, Sanming University, Sanming, Fujian, China

*Dear Editor,*

We have read with interest the in-depth study by Zhang *et al*^[1]^ of the global burden of early-onset type-2 diabetes mellitus (T2DM), and diabetes-related chronic kidney disease (CKD-T2DM), in persons aged 15–39 years. The authors apply join point regression, age–period–cohort modeling and ARIMA forecasting, which show differences in disease trends. Nonetheless, we would like to bring to attention an important aspect that deserves consideration in the understanding of the pathogenesis and prevention of early-onset metabolic disorders, namely, oral microbiome dysbiosis and periodontal health.

The investigators note that while high BMI is the major risk factor (57.2% for T2DM, 51.3% for CKD-T2DM burden), recent evidence indicates that subgingival plaque microbiota changes might be an under-recognized modifiable risk factor. New studies reveal that periodontal pathogens like *Porphyromonas gingivalis* can cause systemic inflammation through lipopolysaccharide, resulting in insulin resistance and insulin resistance, metabolic dysregulation^[2]^. Notably, the “oral-gut microbiome axis” hypothesis refers to the translocation of pathogenic bacteria from subgingival biofilm to the intestinal tract by salivary swallowing, which leads to the disruption of intestinal barrier and ultimately metabolic endotoxemia, which is an important mechanism used in the pathogenesis of T2DM^[3]^.

Evidence from clinical studies indicates that patients with T2DM have specific signatures of the oral microbiome, such as lower alpha diversity and greater abundance of pro-inflammatory taxa^[4]^. Importantly, the severity of periodontal disease was linked to HbA1c levels and insulin resistance indicators, suggesting a bidirectional relationship between oral health and glycemic control. The 2017 World Workshop on the classification of periodontal diseases laid down criteria for the staging and grading of periodontitis that could be incorporated into frameworks for risk stratification of T2DM, especially in younger age groups, where preventive measures can work the best.

Due to the forecasting models of the study showing a 34.94% increase in the global burden of T2DM by 2036^[1]^, it may improve the accuracy of future epidemiological assessments related to T2DM risk if oral health parameters are included. The Global Burden of Disease database could incorporate probing depth, clinical attachment loss, and BOP (Bleeding on Probing) as periodontal health indicators, in addition to the usual metabolic risk factors, in future rounds. This integration will not only elucidate the etiology of early-onset T2DM but will also inform the development of multidisciplinary prevention strategies targeting oral-systemic health connections.

Moreover, from a public health view, the promotion of oral hygiene and periodontal care in youth may be a cost-effective adjunct to obesity prevention. Improving oral health is possible through increased oral hygiene and periodontal practices. In contrast to bariatric surgery or drug treatments, improving oral health is a relatively safe, noninvasive, and applicable option in various settings and places, especially in low- and middle-SDI countries that bear the brunt of the disease burden.

In summary, we congratulate the authors for their thorough analysis and encourage the researchers to explore the oral microbiome and its relationship with metabolic health in other studies on early-onset T2DM. Incorporating oral health aspects into global disease surveillance may bring new prevention approaches while contributing to the WHO’s Global Diabetes Compact aim.

## Data Availability

The data that support the findings of this letter are derived from published literature cited in the references. No original data were generated or analyzed in this study.

## Research registration unique identifying number (UIN)

This letter did not involve human subjects and was based on retrospective data analysis/literature review. Hence, research registration was not applicable.
